# Metallic Antibacterial Surface Treatments of Dental and Orthopedic Materials

**DOI:** 10.3390/ma13204594

**Published:** 2020-10-15

**Authors:** Rushui Bai, Liying Peng, Qiannan Sun, Yunfan Zhang, Lingyun Zhang, Yan Wei, Bing Han

**Affiliations:** 1 Department of Orthodontics, Peking University School and Hospital of Stomatology & National Engineering Laboratory for Digital and Material Technology of Stomatology & Beijing Key Laboratory of Digital Stomatology, 22 Zhongguancun South Avenue, Haidian District, Beijing 100081, China; bairushui@163.com (R.B.); pengliying@bjmu.edu.cn (L.P.); jelena1023@163.com (Q.S.); bdzj_yunfan@163.com (Y.Z.); changlyht@163.com (L.Z.); 2 Department of Geriatric Dentistry, Peking University School and Hospital of Stomatology, 22 Zhongguancun South Avenue, Haidian District, Beijing 100081, China

**Keywords:** metallic agents, surface treatment, antibacterial, dental materials, orthopedic materials, surface modification, coating

## Abstract

The oral cavity harbors complex microbial communities, which leads to biomaterial-associated infections (BAI) during dental and orthopedic treatments. Conventional antibiotic treatments have met great challenges recently due to the increasing emergency of drug-resistant bacteria. To tackle this clinical issue, antibacterial surface treatments, containing surface modification and coatings, of dental and orthopedic materials have become an area of intensive interest now. Among various antibacterial agents used in surface treatments, metallic agents possess unique properties, mainly including broad-spectrum antibacterial properties, low potential to develop bacterial resistance, relative biocompatibility, and chemical stability. Therefore, this review mainly focuses on underlying antibacterial applications and the mechanisms of metallic agents in dentistry and orthopedics. An overview of the present review indicates that much work remains to be done to deepen the understanding of antibacterial mechanisms and potential side-effects of metallic agents.

## 1. Introduction

The oral cavity, containing distinct microenvironments, hosts diverse microbial species including bacteria, archaea, protozoa, fungi, and viruses. Oral bacteria are usually harbored in the oral cavity in the form of biofilms and plaques [1]. The two most common diseases in dentistry, namely dental caries and periodontal diseases, are mainly caused by bacterial plaques [2]. Most dental and maxillofacial treatments are exposed to various bacteria, which could easily accumulate on the surfaces of dental and orthopedic materials [3]. Bacterial infections may result in undesirable complications and an additional burden to patients and doctors [4]. For example, enamel demineralization caused by dental plaques is a common complication of orthodontic treatments [5]. Moreover, the oral biofilm is one of the risk factors of dental implant treatments, associated with peri-implant diseases [6] and could endanger the success of scaffolds in bone restauration [7]. Conventional systemic or local antibiotic treatments are insufficient to handle biomaterial-associated infections (BAI) now, for the abuse of antibiotics in recent decades has led to increasing drug-resistant bacteria [8]. To tackle BAI efficiently, it is necessary to develop better antibacterial dental and orthopedic materials. Among diverse strategies reinforcing the antibacterial property, the surface treatment of materials is currently an area of intensive interest.

Surface treatments could be processed by two main approaches, namely surface modification and coatings [2,9,10]. Surface modification emphasizes the very structure of modified materials, while coatings refer to developing an additional layer on the surface of a substrate [11]. Treated surfaces possess the abilities of inhibiting bacterial adhesion and killing bacteria in contact (passive surfaces), or releasing bactericidal agents and killing bacteria around surfaces (active surfaces) [12].

Implementing surface treatments mainly relies on various antibacterial agents, such as antibiotics [13], non-antibiotic organic antimicrobial agents [14,15], and inorganic antimicrobial agents (e.g., metals and alloys) [11] to achieve bacteriostatic or bactericidal effects. In recent decades, the speed of discovering and producing new effective antibiotics can no longer meet the clinical demand because of the rapidly increasing number of drug-resistant even multidrug-resistant bacteria [16]. Metallic agents endow materials with a low potential to develop bacterial resistance and using metallic agents as alternatives of antibiotics attract much interest now. Moreover, as inorganic substances, metallic agents show chemical stability and protracted action, which are different from traditional organic agents [17]. Due to their excellent broad-spectrum and lasting antibacterial effects, as well as relative biocompatibility with the host, much attention has been paid to antibacterial application and mechanisms of metallic agents in the field of dental and orthopedic material surface treatments. The present review focuses on antibacterial metallic agents used in dentistry. The treated substrate, treating techniques, action against biofilms, and results from these researches are summarized (Table 1). In the following sections, an appraisal of the possible antibacterial mechanisms, antimicrobial assay, biocompatibility, and potential application of these metallic agents is given, along with detailed examples drawn from the literature.

## 2. Chemical Treatments with Metallic Agents

Metallic agents could be synthesized into different sizes, including macro-scale, micro-scale, and nano-scale. Metallic nanoparticles (NPs) are defined as clusters of atoms ranging from 1 nm to 100 nm [47]. High surface-area-to-volume ratio enables NPs special size-related properties different from bulk metals, e.g., better antimicrobial activity under lower concentrations [21,48].

### 2.1. Silver (Ag)

Ag is a nonspecific biocidal agent, exhibiting broad-spectrum bactericidal activities, and could render resistant bacteria to regain antibiotic susceptibility [49]. Considered as a Lewis acid, Ag tends to react with a Lewis base, such as biomolecules containing phosphorous (P) and sulfur (S). The reaction of Ag with P and S, major components of bacterial cell membrane, DNA and proteins, could indicate antibacterial property of Ag [50]. Among different Ag forms, Ag^+^ possess the highest antibacterial activity. Ag has been widely studied in surface treatments of dental and bone implants. Cheng et al. fabricated nanotubular structures loading Ag and Sr on Ti surfaces. They attained long-lasting and controllable release of Ag, resulting in anti-adherent and bactericidal activities against MRSA and *E. coli* [18]. In orthodontics, surface modification and coatings could be used to prevent dental plaque accumulation and dental caries during treatment. Mhaske et al. found that compared to uncoated wires, stainless steel and nickel-titanium archwires coated with Ag showed the anti-adherent effect against *L. acidophilus* [19].

**Figure 1:**
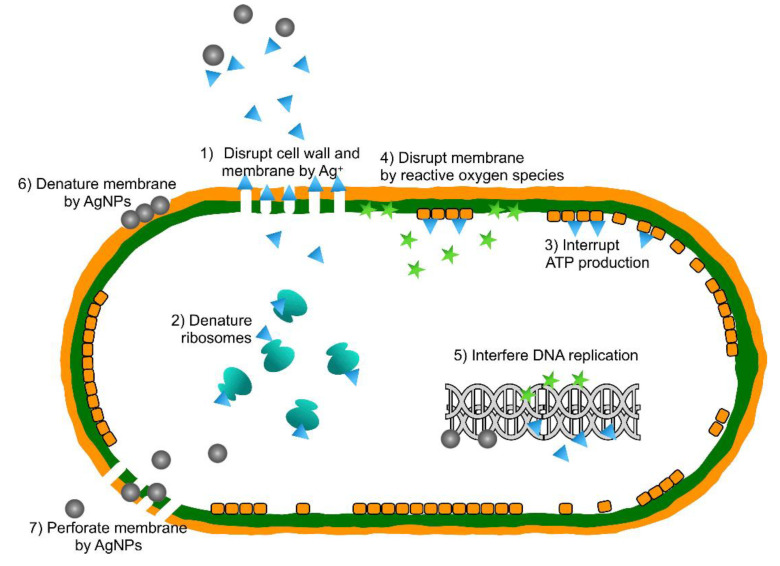
The possible antibacterial mechanisms of AgNPs. Reproduced with permission from ref [53].

Compared to bulk Ag, the nano-scale size makes AgNPs remarkably antibacterial [51], even at a low concentration (Figure 1). Different structural factors could affect the antibacterial property of AgNPs, including surface chemistry, shape, and size, which is clearly elucidated in the review of Tang et al. [52]. Adding AgNPs in implant coatings is an emerging field of research [53]. Zhu et al. immobilized AgNPs on the SLA surface of Ti substrate, which exhibited excellent bactericidal activity against *F. nucleatum* and *S. aureus* [20]. Besinis et al. fabricated a combination of Ag, TiO_2_ and HA nanocoating on titanium alloy (Ti6Al4V). Application of the surface successfully interrupted *S. sanguinis* growth and reduced biofilm formation on implants [21]. AgNPs were also widely used in surface treatments of orthodontic appliances [54]. Hernández-Gómora et al. modified orthodontic elastomeric modules (OEM) with AgNPs and the results showed that treated surface inhibited the growth of *S. mutans*, *L. casei*, *S. aureus,* and *E. coli* [22].

Combining AgNPs with polymers to develop hybrid surfaces can achieve extra bioactive capabilities, such as synergetic antibacterial activity, controlled release of agents and environmental sensitivity. Jin et al. adopted electroplating and ultraviolet reduction technique to modify Ti surface with AgNPs and GO. The multiphase coating showed anti-adherent and antibacterial performance against *P. gingivalis* and *S. mutans*, due to the synergetic effect of AgNPs and GO [23]. Thukkaram et al. loaded AgNPs on an amorphous hydrocarbon matrix to create nanocomposite coatings. This treated matrix could control the release of silver ions to regulate the antibacterial property of the produced coatings [24]. Yang et al. prepared AgNPs within PNIPAAm on glass surfaces to gain “smart” antibacterial activity in response to the change of environmental temperature. The processed surface attached and killed *E. coli* by AgNPs at 37 °C and released dead bacteria at 4 °C because of swollen PNIPAAm chains [25].

### 2.2. Zinc (Zn)

Zn is a transition metal element. The divalent cation, Zn^2+^, also called free Zn, dose not trigger redox reactions under physiological conditions. Zn^2+^ tends to bind to nitrogen and sulfur atoms in histidine and cysteine residues of proteins, leading to little existence of free Zn [55]. Zn^2+^ was reported to possess comparatively higher antibacterial property but less damaging to DNA or the immune system compared to Ag^+^ [28]. Yu et al. used plasma immersion ion implantation to co-implant Zn^2+^ and Mg^2+^ on titanium dental implant surfaces. They found Zn^2+^ could certainly inhibit the growth of oral anaerobic bacteria, including *P. gingivalis*, *F. nucleatum*, and *S. mutans* [26]. Zhao et al. developed Zn/Sr-doped titanium dioxide microporous coating (MT-Zn/Sr) via microarc oxidation on Ti implant surfaces that inhibited the colonization and proliferation of *S. aureus* [27]. On Mg alloy AZ31, Zou et al. loaded Zn^2+^ on montmorillonite (MMT) via a hydrothermal approach for the sustained release of Zn^2+^. Zn-MMT coating exhibited significant antibacterial activity, inhibiting the growth of *S. aureus* and *E. coli* significantly [28].

**Figure 2:**
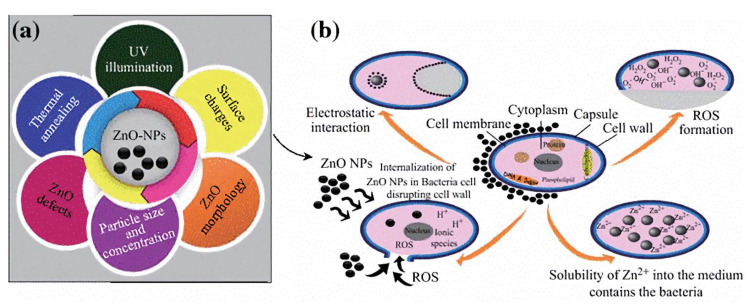
(**a**) Essential parameters of ZnO NPs associated with its antibacterial activity. (**b**) The possible antibacterial mechanisms of ZnO NPs. Reproduced with permission from ref [47].

Zinc oxide (ZnO) is a semi-conductor exhibiting a high bandgap of 3.4 eV and binding energy of 60 meV, which contributes to its unique optical and electrical properties [56]. ZnO also possesses the highest photocatalytic activity among all the inorganic photocatalytic materials [57]. It exhibits broad-spectrum antibacterial activity, especially in nano size (Figure 2) [47]. Li et al. implemented nano ZnO and isocyanate (ISO) resin dual layered modification on 3Y-ZrO_2_ ceramic implants. The ZnO-ISO modified surfaces are endowed with antibacterial activity against *S. aureus* and *E. coli* [29]. Li et al. constructed a hybrid coating consisting of ZnO, SiO_2_, and polystyrene-acrylic acid (PSA) nanoparticles on Ti surfaces. The coating exhibited excellent antibacterial activity against *P. aeruginosa*, *S. aureus,* and *E. coli* [30].

### 2.3. Titanium (Ti)

Ti is usually used as an antibacterial agent in the speciation of TiO_2_, with rutile or anatase crystalline structures. Similar to ZnO, TiO_2_ is also a photocatalyst which could achieve antibacterial property by photocatalytic disinfection [58]. The rutile structure is more thermodynamically stable compared to the anatase structure, while the latter is more photoactive and could be converted to rutile at more than 900 °C. Hence, the crystalline structure of TiO_2_ influences its photocatalytic property significantly [59,60]. In particular, TiO_2_ is a promising agent due to its superior photoreactivity, chemical stability and low toxicity. It can still maintain most of catalytic activity after repeated use [61]. Liu et al. fabricated nanostructured TiO_2_ via atomic layer deposition on Ti implants and concluded that coatings with moderate surface energy showed promising antibacterial activity against *S. aureus*, *E. coli*, and MRSA [31]. Kuroiwa et al. coated TiO_2_ on an autopolymerizable orthodontic acrylic resin and irradiated TiO_2_ by ultraviolet A light. Antibacterial effects were discovered against early colonizers (*S. gordonii*, *S. oralis ATCC*, *S. oralis GTC*, *S. sanguinis*, and *S. mitis*) and cariogenic species (*S. mutans* and *S. sobrinus*) [32]. Chun et al. fabricated a TiO_2_ coating on the stainless-steel orthodontic wires via the sol-gel method and got the antiadherent and bactericidal activity against *S. mutans* [33].

**Figure 3:**
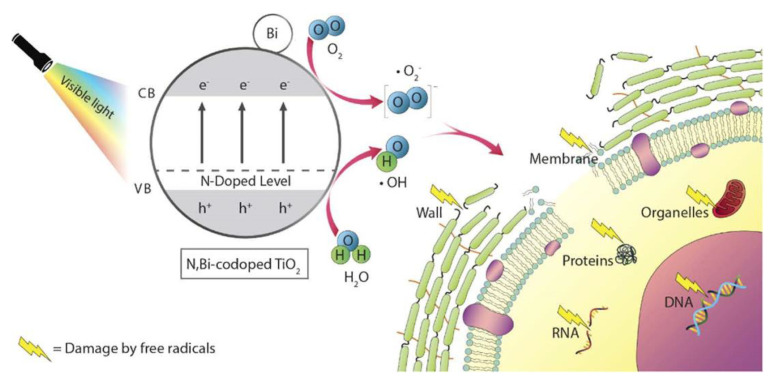
The possible mechanisms of photocatalytic antibacterial activity of N, Bi-codoped TiO_2_. Reproduced with permission from ref [34].

TiO_2_ surfaces can be irradiated by visible light or in the absence of light after proper modification, which solves the problems brought by ultraviolet (UV) irradiation like carcinogenic potential [7]. To shift the band gap of TiO_2_ into the visible light region, Nagay et al. incorporated nitrogen and bismuth into a TiO_2_ coating on Ti implant surfaces. Biofilm formation of *S. sanguinis* and *A. naeslundii* was interrupted by the coating in darkness, and the efficiency was strengthened under visible light (Figure 3) [34]. Antibacterial effects were observed when H_2_O_2_ was catalytic decomposed on TiO_2_ particles in the absence of light, named dark catalysis. Utilizing this phenomenon, Wiedmer et al. created sol-gel derived anatase TiO_2_ coating on porous ceramic scaffolds and successfully obstructed *S. epidermidis* biofilm development in the presence of 3% H_2_O_2_. They further found that TiO_2_ coatings pretreated with 30% H_2_O_2_ could preserve some of the oxidative property even without an oxidative agent [7].

### 2.4. Copper (Cu)

**Figure 4:**
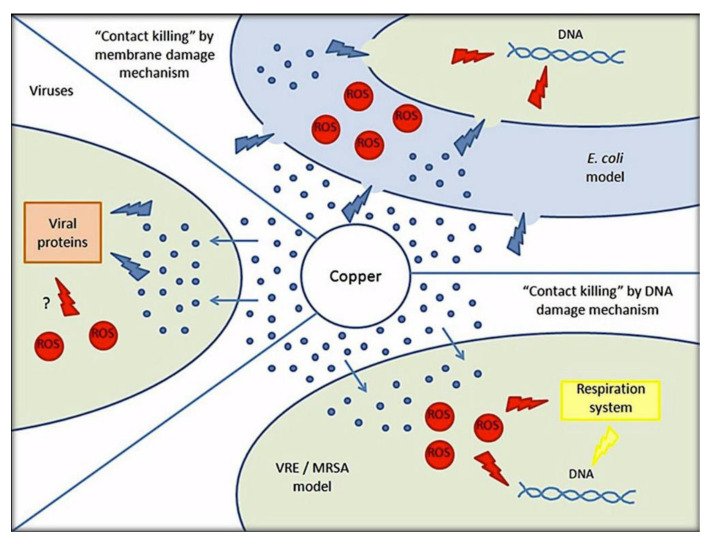
Possible “Contact killing” mechanisms of Cu against bacteria, fungi and viruses. Reproduced with permission from ref [63].

Cu was recognized as the first effective metallic antimicrobial agent by the United States Environmental Protection Agency (EPA) in 2008, possessing wide spectrum antimicrobial properties against bacteria, fungi, and viruses (Figure 4) [62]. Cu exerts antimicrobial activity mainly by contact killing. This phenomenon relies on three physiochemical properties of Cu, including oxidation in ambient conditions, good solubility of Cu oxidizes in the aqueous phase and release of Cu ions by the oxides. Moreover, the soft ionic character and the thiophilicity endow Cu ions with antibacterial activity [63]. In the orthopedic application, Cu is used to coat the ultra-high molecular weight polyethylene (UHMVPE), a promising material for the prosthetic joint. Wu et al. detected excellent dark bactericidal activity of the coating with almost 100% reduction in bacterial number within a short time [35]. Dong et al. modified the stainless steel (SS) surface with a multilayer, containing a nano-crystalline (Fe, Cr, Ni)_3_N deposition layer, a unique Cu-containing co-deposition γ’-M_4_N (M = Fe, Cr, Ni, Cu) layer, and a Cu/N supersaturated phase layer. This modification rendered the SS surface with both quick and durable bactericidal effect [36]. Huang et al. created a chitosan–gelatin (CSG) nanocomposite coating containing Cu via an electrophoretic deposition method on dental implants. The antibacterial activity was positively changed with the concentration of Cu [37]. Rosenbaum et al. fabricated a coating with Cu nanocubes inserted in TiO_2_ nanotubes on Ti substrates. The complete death of *E. coli* and *S. aureus* reflected the high bactericidal property of the coating [38]. Like other metallic agents, Cu in the form of nanoparticle (CuNPs) is also used to develop dental and orthopedic materials with better antibacterial activity. Liu et al. immobilized CuNPs on PEEK implants via the magnetron sputtering technique. Except for the direct bactericidal effect, they also found the indirect immunomodulatory antibacterial effect on the CuNPs coating against MRSA [39].

Cu could also be bound with a tripeptide, named glycine-histidine-lysine (GHK-Cu), to exert biomedical effects. GHK-Cu was a protective and regenerative ingredient discovered in human plasma albumin in 1973, for example, it could reduce free radical damage and inflammation and stimulate wound healing [64,65]. Studies in vivo and vitro found GHK-Cu possessed promising potential to promote bone defects regeneration, for it could enhance the proliferation of human mesenchymal stem cells and increase the attachment of osteoblastic cells [40]. Ning et al. loaded GHK-Cu on a mesoporous silica nanoparticles drug delivery coating on the Ti substrate. They found the coating achieved osteogenic enhancement, antibacterial activity and cytocompatibility simultaneously by PH-controlled releasing of Cu ions [40]. However, not all the researchers agreed with the biocompatibility of GHK-Cu and some of them have implicated the toxicity of Cu for human cells, like hepatocytes [66]. There was a study suggesting that it is not GHK-Cu, but Cu-free GHK that had positive effects on osteoblasts, while GHK-Cu inhibited osteoblastic alkaline phosphatase activity and osteocalcin secretion [67].

### 2.5. Magnesium (Mg)

Mg is a biodegradable, biocompatible and antibacterial metal with capacity to increase osteoblast activity [68]. Feng et al. investigated the antibacterial activities of pure Mg and ZK60 alloy (Mg—6.0 wt % Zn, 0.5 wt % Zr) in Luria−Bertani (LB) medium. Complete elimination of bacteria was achieved in both pure Mg and ZK60 alloy in 24 h, and it was the synergetic actions of Mg and alkalinity ions instead of either one of them or Mg(OH)_2_ that contributed to this biocidal effect [69].

Recent studies have tested the performance of Mg coatings used on implant surfaces. Zaatreh et al. fabricated fast corroding Mg-based coatings on Ti samples and biofilms of *S. epidermidis* decreased significantly without hindering osteoblast viability [41]. Similarly, Zhao et al. constructed Mg-doped TiO_2_ coatings on Ti surfaces and found evenly distributed Mg inhibited bacterial colonization and growth, promoting osteogenesis simultaneously [42].

MgO has also been used as antibacterial agents for biomaterial modification. Wetteland et al. found MgO NPs coatings with 200 μg/mL MgO NPs possessed dual bioactivities, namely antibacterial adhesion and promoting bone marrow derived mesenchymal stem cells proliferation [70]. Coelho et al. added MgO in hydroxyapatite to produce a granular bone substitute. This material successfully inhibited *S. aureus* and *E. coli* growth and biofilm formation, and the antibacterial effect was proportional to concentration of MgO [43].

### 2.6. Other Metallic Agents

Apart from the metallic agents mentioned above, some other metal elements, such as gold (Au), tantalum (Ta) and nickel (Ni) also have antibacterial properties and can be used in surface treatments of dental and orthopedic materials.

Bulk Au is known to be chemically inactive. However, Zheng et al. found gold nanoparticles (AuNPs) can be conferred antimicrobial activity through precise control of their size down to nanoclusters dimension (typically less than 2 nm) [71]. Compared to other metal nanoparticles, AuNPs do not release heavy metal ions in biological fluids and have negligible toxicity, which indicates its relative biosafety [72]. Zheng et al. constructed a 4,6-diamino-2-pyrimidinethiol (DAPT)-conjugated AuNPs coating on various biomedical device substrates. The coatings performed outstanding antibacterial efficiency against pathogenic Gram-negative bacteria and even MDR pathogens and maintained good biocompatibility [44].

**Figure 5:**
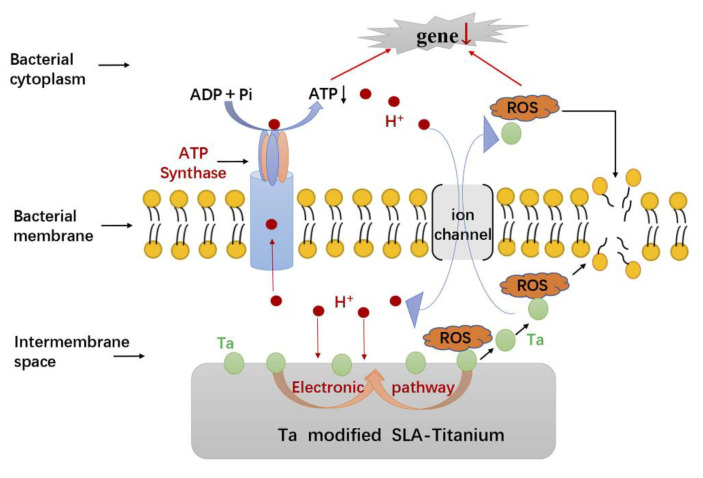
The possible antibacterial mechanisms underlying the antimicrobial activity phenomenon of SLA-Ta surface. Reproduced with permission from ref [45].

Tantalum (Ta) is also a potential antibacterial agent which can hinder biofilm formation. Zhang et al. modified Ti implant surfaces with Ta and observed excellent antibacterial activity against *F. nucleatum* and *P. gingivalis* (Figure 5) [45].

Nickel (Ni) has also been reported to have antibacterial activity. Tested on six species of bacteria, Ni nanoparticles (NiNPs) significantly decreased colony forming unit numbers of bacteria [73]. A study compared antibacterial effectiveness of three types of Ni compounds, and suggested the order as NiCl_2_ > NiNPs > NiO-NPs [74]. Figueroa et al. synthesized Cu-, Ni- and bimetallic Cu-Ni-NPs. They found NiNPs and Cu-Ni NPs possessed only bacteriostatic activity, while CuNPs showed bactericidal activity against *S. aureus, E. coli,* and *S. mutans* [46].

### 2.7. Antibacterial Metal Alloys

Most of the aforementioned substrate getting modification contained only one ingredient, such as titanium or stainless steel. One metallic agent sometimes has one or more drawbacks for application in biomaterials, which stimulates researches about antibacterial metal alloys. Ti and its alloys are one of the most widely used materials in dental and orthopedic materials [75]. Ideal Ti alloys should be multifunctional with antibacterial and osseointegrating activities, biocompatibility as well as high corrosion resistance [76]. These above instructions call for new Ti alloys. Ma et al. developed a copper-titanium alloy (Ti-5Cu), consisting of α-phase matrix and intermetallic compound Ti_2_Cu. This alloy exhibited excellent antibacterial effects via release of Cu ions, and showed better mechanical properties, corrosion resistance and biocompatibility as well [77]. Apart from Ti alloys, Mg alloys also attract attentions of scientists due to their good biodegradable, mechanical and biological properties. Li et al. developed Mg-Cu alloys with different Cu contents and indicated the Mg-Cu alloy with 0.25 wt% Cu had the highest antibacterial effect against MRSA [78].

Antibacterial metal alloys also usually need surface treatments as supplements. Metallic agents could be used for antibacterial surface modification and coatings of metal alloy surfaces, as well. For instance, Zhao et al. fabricated a zirconium dioxide (ZrO_2_) film on the surfaces of magnesium-calcium (Mg-Ca) and magnesium-strontium (Mg-Sr) alloys to deal with their rapid degradation [79].

## 3. Antibacterial Mechanisms of Metallic Agents

Although the exact antibacterial mechanisms of metallic agents are not completely illuminated and still controversial, scientists have proposed several hypotheses and certified some of the antibacterial actions. Donor atom selectivity, reduction potential and speciation are considered three main pertinent chemical determinants of antibacterial properties of metallic agents [80]. Binding among metal ions and bacterial donor molecules could result in bacteriostatic or bactericidal effects. Atomic structures of metals lead to an order of preference for bacterial donor ligands. Interestingly, most of the aforementioned metallic agents contain transition metal elements. Further, the order named Irving–Willams series describes the affinity to ligands of divalent transition metal ions of the fourth period [81]. Reduction potential influences the reactivity of metals, and it is reported that the antibacterial activity of various redox-active metal ions approximately correlates with their standard electrode potentials [82]. Speciation here refers to the existing chemical species and their proportions of metals, which influences the reactivity and solubility of metals. Previous studies suggested that the speciation of a metal, rather than its concentration, play a crucial role for its antibacterial properties [83].

Despite diverse physicochemical properties, different metallic agents could exert antibacterial activities through similar approaches, in brief, disruption or disfunction of cell membrane, interruption of signal transduction, damage of proteins or DNA, oxidation by reactive oxygen species (ROS) and leakage of intracellular contents of bacteria, etc. [80,84].

Antibacterial actions begin from the cytoplasmic membranes of bacteria. Metal cations released in solution, such as Ag^+^ and Zn^2+^, can be attracted to the negatively charged cell membranes of bacteria. The adhered ions consequently interfere with the charge balance and interact with the phospholipid bilayer on surfaces of cell membranes, altering permeability of bacterial membranes [85]. Apart from releasing metal ions, some metal nanoparticles, like Ag NPs, can also penetrate bacteria directly, causing structural and functional damage on cell membranes [53]. Enhanced permeability and damaged membranes induce the leaking in of extracellular contents, leaking out of cytoplasm, or even bacteriolysis [86].

After entering the bacterial cells, metallic agents are capable of further interacting with several molecules and structures inside cells, including DNA, enzymes, proteins, ribosomes and so on. Metallic agents can interrupt DNA replication and cell reproduction by interacting with sulfur and phosphorus, which are vital parts of DNA [53,87]. Metal NPs are able to rapidly bind with enzymes or proteins owing to their small diameters and reduce the activities of various enzymes, resulting in metabolism disorders [88]. AuNPs was also found to inhibit t-RNA binding to ribosome subunits, interrupting production of proteins [89]. All the disturbances mentioned above will act to accelerate the death of bacteria.

Metal ions destroy the mitochondrial electron transport chain of bacteria via deactivation of respiratory enzymes, leading to disturbed ATP production and ROS generation [90]. ROS refer to single-electron reduction products of oxygen, including superoxide anion (O^2−^), hydroxyl radical (OH^−^), and hydrogen peroxide (H_2_O_2_) [91]. ROS generation is the most common and widely accepted mechanism for the antibacterial activity of several metallic agents, such as Ag, ZnO, TiO_2_ and Ta [45,47,61,88,92]. As photocatalytic agents, ZnO and TiO_2_ have common ROS generation that is different from other metallic agents. The ultraviolet or visible light with sufficient photon energy could excite electrons transition and the generation of positively charged holes on ZnO or TiO_2_ surfaces. Electrons and holes participate in redox reactions with water or hydroxide ions to produce ROS [93,94]. ROS exert bactericidal effects by cutting off the chemical bonds of organic substance in bacteria. For instance, negatively charged OH^−^ could not cross the cell membrane, but could aggregate on its surface and denature cell membrane of bacteria. On the contrary, H_2_O_2_ could penetrate and damage the cell membrane, as well as destruct DNA and proteins inside bacterial cytoplasm [23,34,92]. Among metal agents, gold nanoparticle is an exception for its antibacterial activity that is independent of ROS generation, indicating weaker antibacterial property but better biocompatibility to mammalian cells [89].

The antibacterial mechanisms involve a wide range of molecules and physiological processes in bacteria, which guarantees effective bactericidal activities of metallic agents. These multi-process interactions may also account for the low potential for metallic agents to develop bacterial resistance.

## 4. Potential Toxicity of Metallic Agents

Considering biocompatibility, some scientists are concerned about the toxicity of metal ions and nanoparticles [95]. The impact of Ag on human tissues affects its biomedical application. Recent studies suggested that the cytotoxicity of nano- and micro-sized Ag particles was mainly mediated by a size-dependent release of Ag^+^. Ag nanoparticles (50 nm) had stronger cytotoxicity than microparticles (3 μm), and they both decreased cell differentiation and viability of osteoblasts and osteoclasts [96]. AgNPs could also induce cellular nanoparticle uptake and cell stress in human mesenchymal stem cells and osteoblasts [97]. Besides, it is reported that AgNPs induce oxidative stress and impair mitochondrial functions of human cells. After the large dose of AgNPs usage, it could be detected in the liver and spleen. AgNPs even have the potential to pass through the blood-brain barrier and accumulate in the brain [98]. In sum, these potential risks of silver agents bring challenges for its usage and demand methods to decrease Ag^+^ release or uptake of AgNPs into human cells.

A recent study suggested that ZnO NPs induced abnormities of ion content and antioxidant system in liver, but no significant toxic effects to other organisms in rats [99]. Compared with other nanometal oxides, nano-TiO_2_ showed lower toxicity. Particularly, Aruoja et al. found nano-TiO_2_ exerted toxic effects by entrapment of cells, rather than dissolution of metal ions [100]. Copper showed toxicity mainly via ions release from materials. CuNPs possessed greater biotoxicity than bulk copper owing to its larger surface area-to-volume and reactivity [101]. A systematic review concluded that CuNPs could cross the blood-brain barrier and possess neuromuscular toxicity to harm the brain, as well as produce toxicity to lung by DNA damage [102].

Cytotoxic effects of AuNPs have also been reported. Soenen et al. found AuNPs induced ROS to reduce cell viability of human cells under higher concentration and disturbed cell proliferation and differentiation by deforming cytoskeleton [103]. Jun et al. analyzed cytotoxicity of AuNPs with different surface-anchored chiral polymers, having identical physicochemical properties except of reverse chirality. Furthermore, they found different extents of cytotoxicity among these molecules, implying the possibility to design various structures to control the biotoxicity of AuNPs [104].

## 5. Discussion

This review focuses on the metallic antibacterial surface treatments of dental and orthopedic materials. But achieving antibacterial effect should be based on the good biocompatibility with human body cells. So, most of the studies of surface treatments recently aim at creating surfaces with both antibacterial property and nontoxicity to human cells [105]. Ideal antibacterial surface modification and coatings should not only possess biocompatibility with no local or systemic toxicity and proven antibacterial effects, but also excellent mechanical properties, as well as easy and inexpensive approaches for manufacture and use [11].

Apart from the combination of metallic agents with non-organic or non-antibiotic organic agents mentioned in Section 2, some studies also tested the combined antibacterial effects of metallic agents and antibiotics. On the one hand, positive results showed synergistic antibacterial property of these two kinds of agents. Sukhorukova et al. loaded gentamicin or a mixture of gentamicin and amphotericin B on the Ag-doped TiCaPCON coating. They observed that Ag could continue to exert antibacterial property after depletion of antibiotics and increase the antifungal activity of antibiotics [106]. There were also studies using systemic antibiotics treatment and local delivery of AgNPs in vitro and in vivo. The results indicated that AgNPs increased antibacterial efficiency of antibiotics, reduced their usage of and shortened their administration time [107,108]. On the other hand, metal ions, such as Cu and Zn ions, could also act as environmental drivers of antibiotic resistance via co-occurrence of metal resistance and antibiotic resistance genes in animal isolates of multidrug-resistant bacteria [109,110]. Additionally, antibiotics have the ability to complex with metal ions, which may inactivate antibiotics. For instance, binding with Cu compromised the activities of some cephalosporins [111].

The potential biotoxicity have also been discussed above, bringing challenges for using antibacterial metallic agents. However, other scientists tested the biocompatibility of metallic agents and found that soluble metallic agents were only in low concentration. Besinis et al. put the silver nanocoating in a modified Krebs-Ringer bicarbonate buffer for 24 h, and the dissolution was less than 0.07% of the coating [21]. To reduce the underlying risk of metallic toxicity, researches try to control metallic agents released to body tissues, which gets desirable outcomes recently. Zhu et al. immobilized AgNPs on substrates and found toxicity on the viability of rat bone marrow mesenchymal stem cells negligible [20]. Cheng et al. loaded Ag and Sr in nanotubular structures for long-lasting and the controlled release of metals, which showed no apparent cytotoxicity [18]. Therefore, it is necessary for future researches to detect the definite effects of metallic agents on human cells, which is essential for their development and applications. The above discussion prompted that it is important to find a therapeutical concentration window for the usage of metallic antibacterial agents by weighting their antibacterial benefits and potential biotoxicity.

Surface treatments with metallic agents are mostly chemical methods. Apart from chemical techniques, killing bacteria physically though nanostructures, namely drug-free strategies, has since become very topical. Nanostructures, such as nanorods, nanofibers, and nanomats, can both repel bacteria and facilitate tissue integration [112,113]. Obviously, chemical and physical methods could not be separated strictly as some techniques may appeal to multiple physical and chemical processes. A current tendency rising now is to combine metallic agents with nanostructures to improve both antibacterial properties and biocompatibility, as well as reduce side-effects [114]. This strategy of developing compound surfaces on dental and orthopedic materials has promising prospects.

## 6. Conclusions

The current review summarizes researches that addressed the potential application of metallic agents for antibacterial surface treatments of dental and orthopedic materials. The area of surface modification and coatings consists of dental or bone implants, orthodontic appliances, bone regeneration scaffolds, and biomedical devices. The underlying antibacterial mechanisms of metallic agents are also discussed, including the disruption of cell membranes as well as denaturation of molecules and structures inside bacteria. ROS generation and their oxidative effects are common in bactericidal actions. Metallic agents are suitable candidates for antibacterial surface treatments as a result of the broad-spectrum antibacterial property, low potential to develop bacterial resistance, relative biocompatibility, and unique physiochemical characteristics. This potential alternative to antibiotics has become the hotspot of researches in recent years, but still needs further studies for their exact antimicrobial mechanisms and toxicity.

## Figures and Tables

**Table 1:** Summary on researches about metallic antibacterial surface treatments of dental and orthopedic materials.

Antibacterial Metallic Agents	Speciation	Treating Components	Treated Substrate	Treating Techniques	Action against Biofilms	Results	Mentioned Antibacterial Mechanisms	Application	Reference
Ag	Ionic	Ag and Sr loaded nanotubular structures	Ti	Anodization & hydrothermal method	MSSA, MRSA, *E. coli*	Controllable release of Ag and Sr; Ag: anti-adherent & bactericidal activities against bacteria; Sr: accelerated filling of bone defects.	Not mentioned	Bone/dental implants	Cheng et al. [18]
	Metallic	Ag	SS, NiTi	Thermal vacuum evaporation method	*L. acidophilus*	Anti-adherent effect against bacteria.	Ag binds to key functional groups of enzymes.	Orthodontic wires	Mhaske et al. [19]
	NPs	Immobilized AgNPs	SLA-Ti	Silver plasma immersion ion implantation	*F. nucleatum,* *S. aureus*	Good defense against multiple cycles of bacteria attack & excellent compatibility with mammalian cells.	Ag^0^ rendered by AgNPs with electron trapping capability disrupts the integrity of bacterial membranes.	Dental implants	Zhu et al. [20]
	NPs	AgNPs, TiO_2_ and nano HA	Ti alloy (Ti6Al4V)	Silver plating, anodization & sintering techniques	*S. sanguinis*	Inhibition of bacterial growth in the surrounding media and biofilm formation on the implant surface, maintaining the HA biocompatibility.	Direct contact toxicity with small but effective slow release of Ag; oxidative stress from free radicals generated by Ag-TiO_2_-HA.	Dental implants	Besinis et al. [21]
	NPs	AgNPs	OEM	Bioreduction of AgNO_3_	*S. mutans,* *L. casei,* *S. aureus,* *E. coli*	Inhibiting growth of bacteria and enhancing physical properties.	AgNPs inhibits theenzymes of the cell respiratory cycle and damages DNA synthesis, leading to cell death.	OEM	Hernández-Gómora et al. [22]
	NPs	AgNPs and GO	Ti	Electroplating & ultraviolet reduction methods	*P. gingivalis, S. mutans*	Excellent antimicrobial ability and anti-adherence performance.	AgNPs causes bacterial DNA damage, interruption of cell signal transduction, oxidative damage of ROS, intracellular contents leakage and dehydrogenase inactivation.	Dental implants	Jin et al. [23]
	NPs	AgNPs loaded a-C:H matrix	Ti	GAS & PE-CVD process	*E. coli,* *S. aureus*	Controlled release of Ag^+^, excellent antibacterial performance and good biocompatibility.	The antibacterial efficacy of AgNPs coating is associated with their ability to release Ag^+^.	Orthopedic implants	Thukkaram et al. [24]
	NPs	AgNPs and PNIPAAm	Glass	One-step photopolymerization method	*E. coli*	“Smart” antibacterial capability to attach, kill, and release bacteria in response to the change in environmental temperature.	AgNPs releases Ag^+^ to affect the metabolism of *E. coli* and weaken the interaction between *E. coli* and the substrate.	Biomedical materials	Yang et al. [25]
Zn	Ionic	Zn^2+^ & Mg^2+^	Ti	Plasma immersion ion implantation	*P. gingivalis,* *F. nucleatum,* *S. mutans*	Inhibition of oral anaerobic bacteria, good osteo-inductivity and proangiogenic effects.	Inhibiting bacterial adhesion and growth by Zn^2+^ release and ROS generation.	Dental implants	Yu et al. [26]
	Metallic	Zn/Sr-doped microporous TiO_2_	Ti	Microarc oxidation	*S. aureus*	Inhibiting bacterial colonization and proliferation with biocompatibility.	Zn^2+^ inhibits bacterial growth via inducing cell lysis and cytoplasmic leakage.	Dental implants	Zhao et al. [27]
	Ionic	Zn-MMT	Mg alloy AZ31	Hydrothermal method	*E. coli,* *S. aureus*	Sustained-release of Zn^2+^, good antibacterial activity, biocompatibility and corrosion resistance.	Zn-MMT leads to severe breakage of bacterial membrane; sustainable release of Zn^2+^ around.	Orthopedic applications	Zou et al. [28]
	Oxide NPs	Nano ZnO & isocyanate resin	3Y-ZrO_2_ ceramics	Thermal spray coating process	*E. coli,* *S. aureus*	Broad-spectrum antibacterial behavior, no obvious noticeable tissue damage in all major organs of mice.	Not mentioned.	Ceramic implants	Li et al. [29]
	Oxide NPs	N-halamine labeled ZnO, silica PSA NPs	Ti	Electrostatic adsorption	*P. aeruginosa,* *E. coli,* *S. aureus*	Excellent antibacterial activity, good biocompatibility toward the preosteoblast.	Making bacterial membranes distorted and incomplete.	Implants	Li et al. [30]
Ti	Oxide NPs	Nanostructured TiO_2_	Ti	Temperature-controlled atomic layer deposition	MSSA, MRSA, *E. coli*	The coating with a moderate surface energy showed relatively promising antibacterial properties and desirable cellular functions.	Photoactivated TiO_2_ destructs bacteria; increased surfaces roughness at the nano-scale limits the number of anchoring points for bacteria.	Orthopedic implants	Liu et al. [31]
	Oxide	TiO_2_	Autopolymerizable acrylic resin	Spin-coating methods	*S. mutans,* *S. sobrinus,* *S. gordonii,* *S. oralis,* *S. sanguinis,* *S. mitis*	Antibacterial effects were discovered against early colonizers and cariogenic species.	TiO_2_ induces hydroxyl radical attack, leading to bacterial cytoplasmic membrane.	Removable orthodontic resin-based retainer	Kuroiwa et al. [32]
	Oxide	TiO_2_	SS	Sol-gel thin film dip-coating method	*S. mutans,* *P. gingivalis.*	Antiadherent and antibacterial properties.	TiO_2_ breaks down the cell wall of bacteria.	Orthodontic wires	Chun et al. [33]
	Oxide	TiO_2_ codoped with nitrogen and bismuth	Ti	Plasma electrolytic oxidation	*S. sanguinis,* *A. aeslundii*	Antibacterial properties in darkness, with a stronger effect after visible-light application, noncytotoxic effect on fibroblast cells.	Photocatalytic effect of TiO_2_ generates ROS to decompose bacterial organic compounds.	Dental implants	Nagay et al. [34]
	Oxide	Sol-gel derived anatase TiO_2_ coating	Porous ceramic scaffolds	Sol-gel derived anatase coating, catalytic decomposition of H_2_O_2_ in dark	*S. epidermidis*	Antibacterial activity, particularly at the early stages of *S. epidermidis* biofilm development, no cytotoxic effects.	Presence of the superoxide anion via dark catalysis of TiO_2_ and a ROS-mediated killing mechanism.	Bone Scaffolds	Wiedmer et al. [7]
Cu	Metallic	Cu	UHMVPE	Low temperature aerosol assisted chemical vapor deposition	*E. coli,* *S. aureus*	Potent dark bactericidal activity with 99.999% reduction in bacterial number within 15 min.	Generated ROS triggers oxidation of unsaturated fatty acid in the cell membrane; proteins and DNA degradate.	Prosthetic joint	Wu et al. [35]
	Metallic	Cu and a supersaturated phase (S-phase)	Austenitic SS	Active screen plasma alloying technology	*E. coli*	Quick bacterial killing rate and durability.	Cu interacts with the thiol groups of bacterial proteins and enzymes to inactivate bacteria.	Medical devices	Dong et al. [36]
	Ionic	Cu-doped chitosan-gelatin nanocomposite coating	Ti	Electrophoretic deposition method	*E. coli,* *S. aureus*	Antibacterial, angiogenic, and osteogenic properties, with low cytotoxicity.	Cu destroys the permeability of bacterial membranes, leading to leakage of bacterial proteins.	Ti-based materials	Huang et al. [37]
	NPs	Cu nanocubes deposited TiO_2_ nanotubes	Ti	Anodic oxidation and pulsed electrodeposition	*E. coli,* *S. aureus*	High bactericidal potential with complete death of bacteria.	Preferential release of Cu^+^ is considerably more toxic to bacteria than Cu^2+^.	Dental implants	Rosenbaum et al. [38]
	NPs	CuNPs	PEEK	Magnetron sputtering technique	MRSA	Direct antibacterial and indirect immunomodulatory antibacterial effects against MRSA.	Contact-killing effect: destroy permeability of bacterial membranes, cell respiration; genetic toxicity.	Implants	Liu et al. [39]
	Ionic	Chitosan loaded with MSN@GHK-Cu (glycyl-L-histidyl-L-lysine-Cu^2+^)	Ti	Electrophoretic deposition	*E. coli,* *S. aureus*	Inhibited adhesion of bacteria but with good cytocompatibility.	Cu^2+^ changes bacterial membrane permeability, induces ROS generation, destroys cell structures and metabolic process.	Orthopedic and dentalimplants	Ning et al. [40]
Mg	Metallic, alloy	Mg or Mg_45_Zn_5_Ca	Ti	Magnetron sputtering	*S. epidermidis*	Antibacterial properties and low cytotoxicity levels.	Corrosion of Mg and its alloys results in shift in pH, killing bacteria by osmotic shock and inhibiting bacterial adhesion.	Implants	Zaatreh et al. [41]
	Ionic	Mg-doped TiO_2_	Ti	Plasma electrolytic oxidation	*S. aureus*	Inhibiting bacterial colonization and growth; promoting osteoblast adhesion, proliferation and differentiation.	Mg^2+^ penetrates bacterial cell walls, degenerates bacterial proteins, abolishes the activity of bacterial synthetase and causes bacteria to lose proliferation ability.	Implants	Zhao et al. [42]
	Oxide NPs	MgO NPs	HA	Ionotropic gelation method	*E. coli,* *S. aureus*	Reduced bacterial growth and biofilm formation in a concentration-dependent manner	Physical membrane damage; non-ROS mediated toxicity; non-Mg^2+^ release toxicity.	Bone substitutes	Coelho et al. [43]
Au	NPs	AuNPs & 4,6-diamino-2-pyrimidinethiol	PS, PVC, PP, PE, PDMS, SiO_2_	Electrostatic self-assembly	*E. coli*, *P. aeruginosa*, *K. pneumoniae*, *S. aureus*, MDR *E. coli*, MDR *P. aeruginosa*, MDR *K. pneumoniae*	Outstanding antibacterial activity against Gram-negative bacteria on a variety of surfaces.	Immobilized AuNPs disrupts bacterial cell membranes.	Medical devices	Zheng et al. [44]
Ta	Metallic	Ta	SLA-Ti	Magnetron-sputtering technique	*F. nucleatum,* *P. gingivalis*	Excellent antimicrobial activity, promoted osseointegration of implants.	Ta inhibits bacterial ATP synthesis, promotes ROS generation and eventually disrupts cellular metabolism.	Dental implants	Zhang et al. [45]
Ni	NPs	Ni or bimetallic Cu–Ni NPs	None	Synthesized in aqueous solution without using stabilizers.	*E. coli*, *S. aureus*, *S. mutans*	Exhibiting only bacteriostatic effect.	Bacteriostatic effect, without bactericidal effect.	Dental materials	Figueroa et al. [46]

Abbreviation: Ag: silver. Sr: strontium. Ti: titanium. MSSA: Methicillin-sensitive *Staphylococcus aureus*. MRSA: methicillin-resistant *Staphylococcus aureus*. *E. coli*: *Escherichia coli*. SS: stainless steel. NiTi: nickel-titanium. *L. acidophilus*: *Lactobacillus acidophilus*. NPs: nanoparticles. SLA: sand-blasted, large grit, and acid-etched. *F. nucleatum*: *Fusobacterium nucleatum*. Ag^0^: neutral metallic silver. *S. aureus*: *Staphylococcus aureus*. TiO_2_: titanium oxide. HA: hydroxyapatite. *S. sanguinis: Streptococcus sanguinis*. OEM: Orthodontic elastomeric modules. AgNO_3_: silver nitrate. *S. mutans: Streptococcus mutans*. *L. casei: Lactobacillus casei*. DNA: deoxyribonucleic acid. GO: graphene oxide. *P. gingivalis: Porphyromonas gingivalis*. ROS: reactive oxygen species. a-C:H: amorphous hydrocarbon. GAS: gas aggregation source. PE-CVD: plasma-enhanced chemical vapor deposition. Ag^+^: silver ions. PNIPAAm: Poly(N-isopropylacrylamide). Zn: zinc. Zn^2+^: zinc ions. Mg^2+^: magnesium ions. Zn-MMT: Zn-loaded montmorillonite. ZnO NPs: zinc oxide nanoparticles. PSA: polystyrene-acrylic acid. *P. aeruginosa: Pseudomonas aeruginosa*. *S. sobrinus: Streptococcus sobrinus*. *S. gordonii: Streptococcus gordonii*. *S. oralis: Streptococcus oralis*. *S. mitis: Streptococcus mitis*. *A. aeslundii: Actinomyces aeslundii*. *S. epidermidis: Staphylococcus epidermidis*. Cu: copper. UHMVPE: ultra-high molecular weight polyethylene. Cu^+^: monovalent copper ions. Cu^2+^: bivalent copper ions. PEEK: polyetheretherketone. MgO: magnesium oxide. PS: polyethylene. PVC: polyvinyl chloride. PP: polypropylene. PE: polyethylene. PDMS: polydimethylsiloxane. SiO_2_: silica. *K. pneumoniae: Klebsiella pneumoniae*. MDR: multi-drug resistant. Ta: tantalum.
